# Magnetic Properties and Strengthening Mechanism of Cu-Bearing Non-Oriented Silicon Steel

**DOI:** 10.3390/ma18184233

**Published:** 2025-09-09

**Authors:** Shi Qiu, Yuhao Niu, Kaixuan Shao, Bing Fu, Haijun Wang, Jialong Qiao

**Affiliations:** 1 College of Design and Engineering, National University of Singapore, Lower Kent Ridge Road, Singapore 119260, Singapore; e1351820@u.nus.edu; 2 National Engineering Research Center of Continuous Casting Technology, China Iron & Steel Research Institute Group Co., Ltd., Beijing 100081, China; 3 Silicon Steel & Sheet Business Division, Xinyu Iron and Steel Group Co., Ltd., Xinyu 338001, China; 4 School of Metallurgical Engineering, Anhui University of Technology, Ma’anshan 243032, China

**Keywords:** non-oriented silicon steel, microstructure, texture, copper precipitate, magnetic properties, mechanical properties

## Abstract

The effects of Cu content on the microstructure, texture, precipitates, and magnetic and mechanical properties of 0.20 mm-thick non-oriented silicon steel (3.0% Si-0.8% Al-0.5% Mn) were systematically investigated using optical microscopy, X-ray diffraction, electron backscatter diffraction, and transmission electron microscopy. The strengthening mechanisms of Cu-bearing high-strength non-oriented silicon steel were further elucidated. Increasing Cu content inhibited grain growth and suppressed the development of the α*-fiber texture in annealed sheets, while promoting the formation of γ-fiber texture. As a result, the P_1.0/400_ and B_50_ values deteriorated. The P_1.0/400_ and B_50_ values of 1.47% Cu non-oriented silicon steel were 13.930 W/kg and 1.614 T, respectively. However, due to the solid solution strengthening effect of 0.5% Cu and partial precipitation strengthening, the R_p0.2_ increased by 43 MPa. After aging treatment at 550 °C for 20 min, the P_1.0/400_ values of the aged sheets slightly increased, while the B_50_ values remained almost unchanged. In the aged sheets containing 1.0–1.5% Cu, clustered Cu-rich precipitates with average sizes of 2.71 nm and 13.28 nm were observed. The crystal structure of these precipitates transitioned from the metastable B2-Cu to the stable FCC-Cu. These precipitates enhanced the R_p0.2_ of the non-oriented electrical steel to 241 MPa and 269 MPa through cutting and bypass mechanisms, respectively. A high-strength non-oriented silicon steel with balanced magnetic and mechanical properties was developed for driving motors of new energy vehicles by utilizing nanoscale Cu-rich precipitates formed through aging treatment. The optimized steel exhibits a yield strength of 708 MPa, a magnetic induction B_50_ of 1.639 T, and high-frequency iron loss P_1.0/400_ of 14.77 W/kg.

## 1. Introduction

To mitigate the global energy crisis and environmental degradation, products such as new energy vehicles, drones, and centrifugal compressors, which are representative of clean energy, are evolving rapidly [1,2]. Non-oriented silicon steel, as the core material for manufacturing drive motor cores, requires high magnetic induction, low core loss at high frequencies, and high strength [3,4,5]. Currently, the rotational speed of electric vehicle traction motors is in the range of (2~3) × 10^4^ rpm, and the yield strength of non-oriented silicon steel needs to exceed 440 MPa [6]. At the same time, the development of a new generation of non-oriented silicon steel with high magnetic induction, low core loss at high frequencies, and high strength has become a formidable challenge in both academia and industry [7,8].

The solubility of Cu in ferrite is approximately 2.1%, and it has a low lattice misfit with the BCC matrix. Therefore, tailoring the precipitation behavior of Cu-rich particles has emerged as a promising approach for developing non-oriented electrical steels with superior magnetic and mechanical properties [9,10,11]. Following aging treatment at 550 °C for 25 min, Wang et al. [12] obtained nanoscale copper precipitates with an average size of 3.2 nm, which allowed dislocations in the matrix to cut through the high-density nanoscale copper precipitates via slip. As a result, the yield strength and tensile strength reached 669 MPa and 780 MPa, respectively. Ni et al. [13] proposed that the optimal copper addition in 3.2% Si-1.0% Al non-oriented silicon steel is 0.91%. After annealing, nanoscale B2 Cu-rich precipitates were formed in the matrix, and the dispersed and coherent nanoscale B2 Cu-rich precipitates contributed more to the strength through the cutting mechanism than the larger ε-Cu precipitates, which increased strength through the bypass mechanism. Consequently, the yield strength of the non-oriented silicon steel reached 571 MPa.

Regarding magnetic properties, Wu et al. [14] demonstrated that in non-oriented electrical steels containing 0–0.25% Cu, copper precipitates effectively controlled microstructure and magnetic properties. With increasing copper content, the amount of precipitates increased, and particle coalescence occurred, resulting in a significant reduction in core loss without a noticeable decrease in magnetic induction. Meanwhile, Bian et al. [15] found that in non-oriented silicon steel with copper content ranging from 0.11% to 0.35%, the size of the precipitates remained almost unchanged as copper content increased. However, the number of precipitates significantly increased, and the Cu-rich precipitates inhibited the {111} texture component while promoting the Goss component, thus improving the recrystallization textures and significantly reducing the core loss.

Therefore, this study systematically investigated the influence mechanisms of copper content on the magnetic and mechanical properties of 3.0% Si-0.8% Al-0.5% Mn non-oriented electrical steel. Multiscale characterization techniques, such as optical microscopy (OM), X-ray diffraction (XRD), electron backscatter diffraction (EBSD), and transmission electron microscopy (TEM), were employed to analyze and compare the evolution of microstructure and texture during the normalizing, cold rolling, and annealing processes for non-oriented silicon steel with varying copper contents. Additionally, the precipitation behavior of copper precipitates after aging treatment and the strengthening mechanism of Cu-bearing high-strength non-oriented silicon steel were investigated.

## 2. Materials and Methods

**Figure 1:**
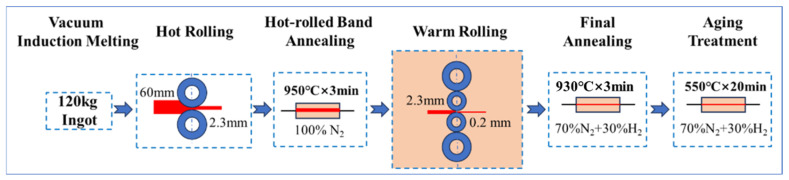
Schematic diagram of the processing route for the experimental steels.

The experimental steels were melted in a 200 kg vacuum induction furnace, and the ingot dimensions were 60 mm (thickness) × 400 mm (width) × 700 mm (length). The main chemical composition is shown in Table 1. In the subsequent research, the test steels were labeled Cu-0, Cu-0.5, Cu-1.0, and Cu-1.5. Hot rolling was performed with 60 mm thick plates reduced to 2.3 mm through 7 passes using a 400 mm rapid hot rolling experimental mill. The reheating temperature of the slab was 1150 °C, and the final rolling temperature was 850 °C. The reduction rate of the seventh pass ranged from 14% to 17%. Hot-rolled band annealing was carried out at 950 °C for 3 min. The hot-rolled bands were pickled with hydrochloric acid to remove the oxide layer, then rolled to 0.20 mm thickness at 120 °C using a 350 mm hydraulic tension cold-warm rolling experimental mill. An annealing treatment was then performed at 930 °C for 3 min. Finally, the annealed sheets were aged at 550 °C for 20 min. A schematic of the process is shown in Figure 1.

Samples were prepared in sizes of 10 mm (rolling direction, RD) × 8 mm (transverse direction, TD). After grinding and polishing, they were etched with 4 vol% nitric acid solution and observed under an optical microscope (Axio Scope A1, ZEISS, Jena, Germany) for microstructure analysis. Microtexture detection was conducted using a field emission scanning electron microscope (SUPRA 55VP, ZEISS, Jena, Germany) equipped with an EDAX OIM electron backscatter diffraction system. The Aztec Crystal 2.1.2 software was used to quantitatively analyze the area fraction and average grain size of specific oriented grains, with more than 500 grains measured for each specimen. A deviation angle of 15° was applied to define a particular texture component. Macrotexture analysis was performed using an XRD (EMPYREAN SERIES 2, Malvern Panalytical, Almelo, The Netherlands) with Co Kα radiation. Orientation distribution functions (ODFs) were calculated from pole figures using X’Pert Texture 1.2 software. For copper precipitate characterization, aged samples were first pre-ground to 70–80 μm, followed by electrolytic thinning using a TenuPol-5 electrolytic twin-jet polisher (5% perchloric acid + 95% ethanol, with a current of 30V and 75 mA at −30 °C) to obtain TEM samples. The precipitation states of the samples were observed using a JEM-F200 TEM (JEOL, Tokyo, Japan) at 200 kV.

The magnetic properties of the annealed and aged samples were measured using a TPS-500M silicon steel measuring instrument (Hunan Linkjoin Technology Co., Ltd., Loudi, China), including magnetic induction B_50_ (5000 A/m, 50 Hz), low-frequency core loss P_1.5/50_ (1.5 T, 50 Hz), and high-frequency core loss P_1.0/400_ (1.0 T, 400 Hz). The standard sample size was 300 mm × 30 mm. To ensure the accuracy of the magnetic properties, the average values of three transverse-direction samples and three rolling-direction samples were used as the final magnetic properties. Tensile strength (R_m_, MPa) and yield strength (R_p0.2_, MPa) were measured using an MTS880 tensile testing machine (MTS, Shanghai, China). The sample size was 130 mm × 20 mm × 0.20 mm, with a gauge length of 50 mm.

## 3. Results and Discussion

### 3.1. Microstructure and Texture

**Figure 2:**
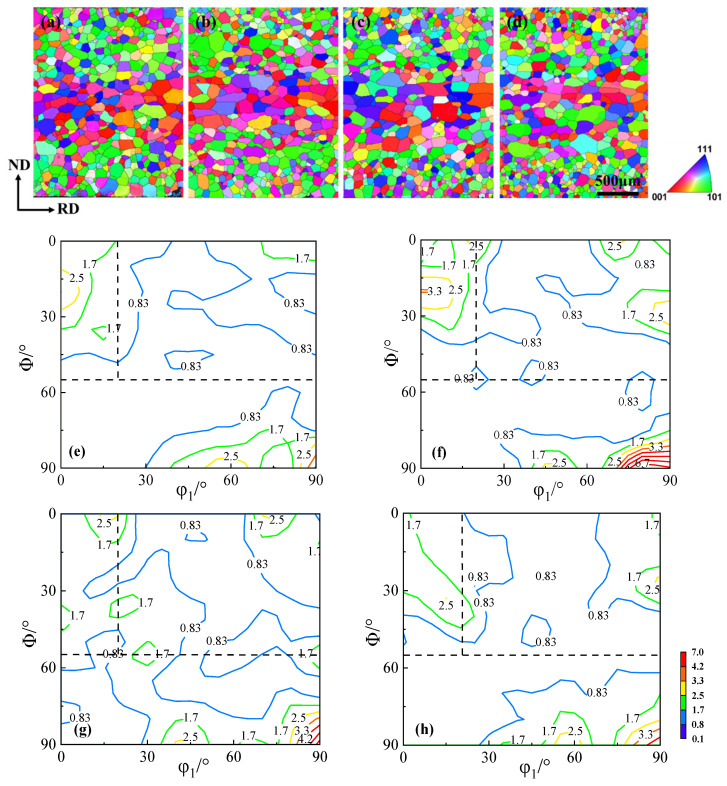
Grain orientation image maps and the φ_2_ = 45° ODF section of the normalized sheets. (**a**,**e**) Cu-0, (**b**,**f**) Cu-0.5, (**c**,**g**) Cu-1.0, and (**d**,**h**) Cu-1.5.

The grain orientation imaging maps along the thickness direction, and the macrotextures of the normalized sheets are shown in Figure 2. The surface grain size of the normalized sheets with different Cu contents is significantly smaller than that of the central layer, with the surface and sub-surface layers predominantly consisting of {110}-oriented grains, while the central layer mainly consists of coarse {100}-oriented grains. With increasing Cu content, the predominant textures of the normalized sheets remain unchanged, but the intensity corresponding to the main textures changes significantly.

**Figure 3:**
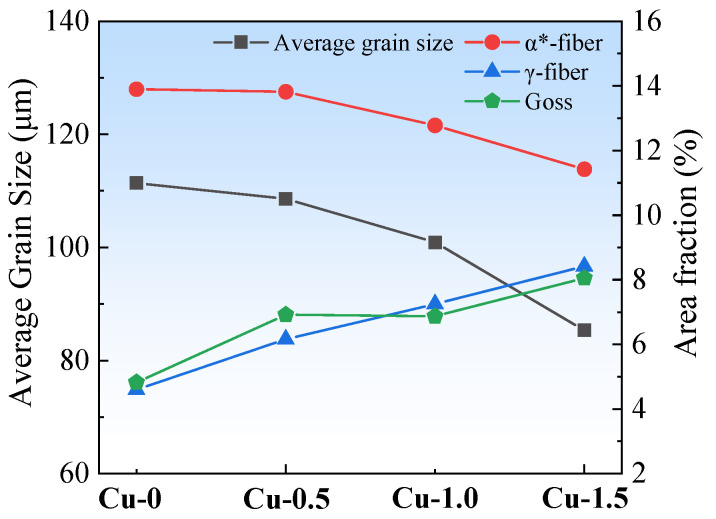
The average grain sizes and the area fraction of the predominant textures of the normalized sheets.

The average grain size and the area fraction of the predominant textures are shown in Figure 3. The right axis represents the area fraction percentages of α*-fiber, γ-fiber, and Goss textures. With increasing Cu content, the average grain size and the area fraction of α*-grains decrease significantly, while the area fraction of γ-grains and Goss grains increases. Copper precipitates pin dislocations and subgrain boundaries, which suppresses grain recrystallization during normalization, thereby promoting grain size homogeneity. As the Cu content increases, the precipitate size grows, enhancing the resistance to grain boundary migration, resulting in grain refinement in the normalized sheets. Therefore, the Cu-1.5 normalized sheet exhibits the minimum average grain size [14].

**Figure 4:**
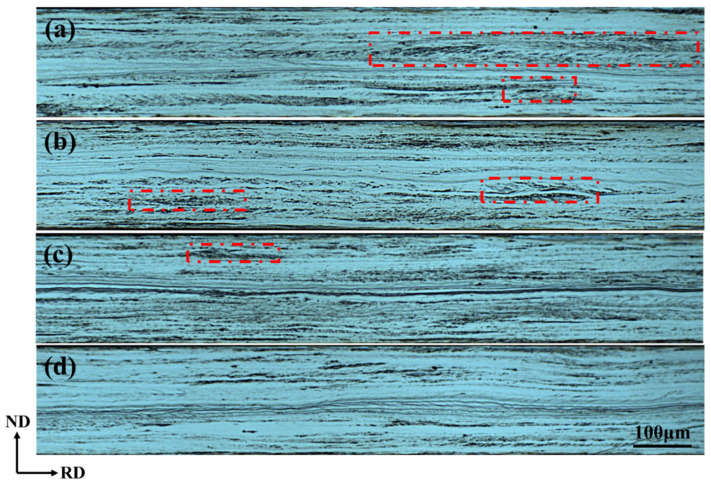
Microstructures of the cold-rolled sheets. (**a**) Cu-0, (**b**) Cu-0.5, (**c**) Cu-1.0, and (**d**) Cu-1.5.

The microstructures of cold-rolled sheets with varying Cu contents, rolled from 2.3 mm to 0.2 mm, are shown in Figure 4. The cold-rolled sheets primarily consist of deformation bands and shear bands with 20–35° inclination to the rolling direction (red dashed box). The grains with high stored energy are more darkly etched due to their higher Taylor factor and flow stress, which resist planar deformation. These grains exhibit a “rough” surface feature, while grains with low stored energy are lightly etched, displaying a “smooth” surface feature [16].

The characteristics of intragranular shear bands in cold-rolled sheets with different Cu contents show significant differences. Abundant shear bands develop in Cu-0 and Cu-0.5 cold-rolled sheets (as indicated by the red dashed lines in Figure 4), whereas fewer shear bands form in Cu-1.0 cold-rolled sheets. The Cu-1.5 cold-rolled sheet shows negligible intragranular shear band formation. This significant difference is attributed to the larger grain sizes in the normalized sheets. According to the study by Liu et al. [17], coarse grains in the normalized sheets significantly promote the formation of intragranular shear bands in the cold-rolled microstructure. Clearly, the Cu-0 and Cu-0.5 normalized sheets have larger grain sizes, which are 111.4 μm and 108.6 μm, respectively. Increasing Cu content progressively reduces the number of shear bands in cold-rolled sheets. Consequently, grain refinement in normalized sheets with higher Cu content suppresses the development of in-grain shear bands during cold rolling.

**Figure 5:**
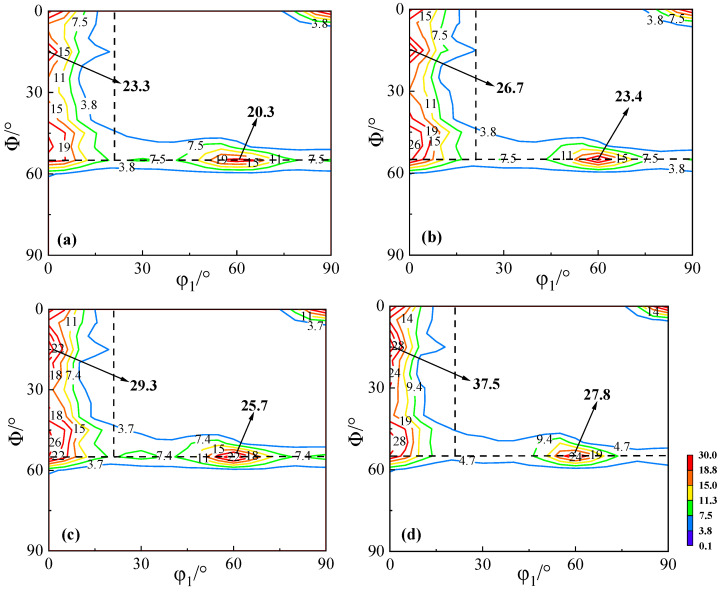
Macro-textures (φ2 = 45° ODF section) of the cold-rolled sheets. (**a**) Cu-0, (**b**) Cu-0.5, (**c**) Cu-1.0, and (**d**) Cu-1.5.

Figure 5 shows the macrotextures of cold-rolled sheets with different Cu contents. The texture types of the cold-rolled sheets are essentially consistent, exhibiting strong α-fiber, γ-fiber, and λ-fiber textures, which are concentrated in {114} <110>, {111} <110>, and {001} <110>, respectively. The intensity of the α-fiber decreases gradually with increasing Cu content, while the intensity of the γ-fiber increases. Since the Cu-0 and Cu-0.5 normalized sheets have larger grain sizes, it is more difficult for the cold-rolled sheets to rotate uniformly to stable orientations during the rolling process. Therefore, the intensities of the α-fiber are 23.3 and 26.7, while the intensities of the γ-fiber are 20.3 and 23.4, respectively. In contrast, for the Cu-1.0 and Cu-1.5 normalized sheets, which have smaller grains, the cold-rolled sheets mainly exhibit elongated deformation structures, and the matrices rotate to stable orientations. As a result, the intensities of α-fiber are 29.3 and 37.5, and the intensities of γ-fiber are 25.7 and 27.8, respectively.

**Figure 6:**
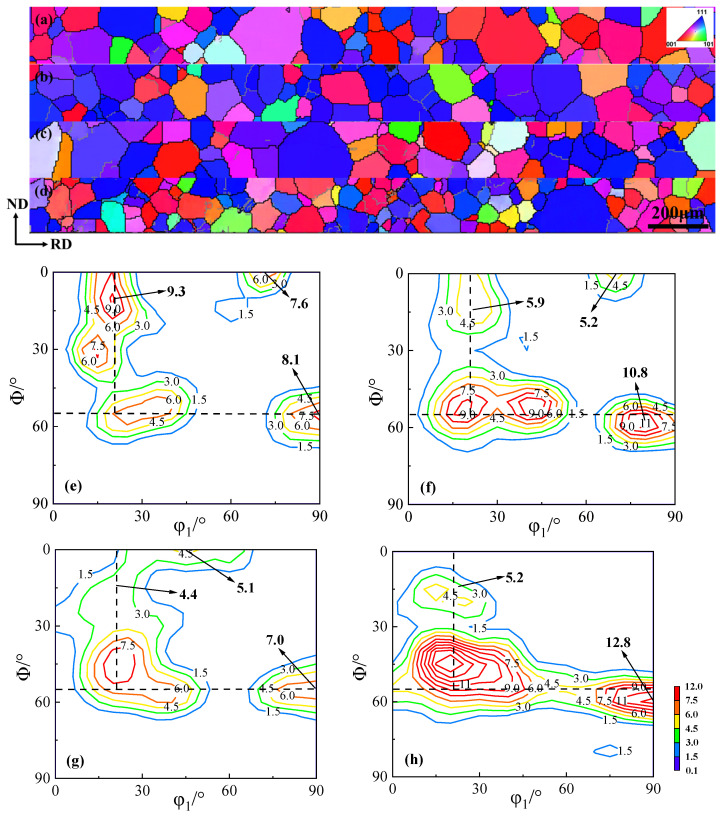
Grain orientation image maps and macro-textures (φ2 = 45° ODF section) of the annealed sheets. (**a**,**e**) Cu-0, (**b**,**f**) Cu-0.5, (**c**,**g**) Cu-1.0, and (**d**,**h**) Cu-1.5.

After annealing at 930 °C for 3 min, the grain orientation image maps and macro-textures of the annealed sheets are shown in Figure 6. The grain size of the annealed sheets significantly decreases as the Cu content increases, but the predominant textures remain similar. The primary grain orientations in the annealed sheets are the α*-fiber, γ-fiber, and λ-fiber, which are concentrated near {114} <481>, {111} <112>, and {100} <012> textures, respectively.

As the Cu content increases in the annealed sheets, the intensity of the unfavorable γ-fiber increases from 6.6 in the Cu-0 annealed sheet to 9.3 in the Cu-1.5 annealed sheet, while the intensity of the favorable α*-fiber decreases from 8.0 to 2.0, and the intensity of the λ-fiber almost disappears. Therefore, the copper precipitates promote the development of the {111} texture and suppress the development of the {100} texture during annealing, resulting in the strongest intensity of γ-fiber in the Cu-1.5 annealed sheet, while the intensities of α*-fiber and λ-fiber are the weakest.

**Figure 7:**
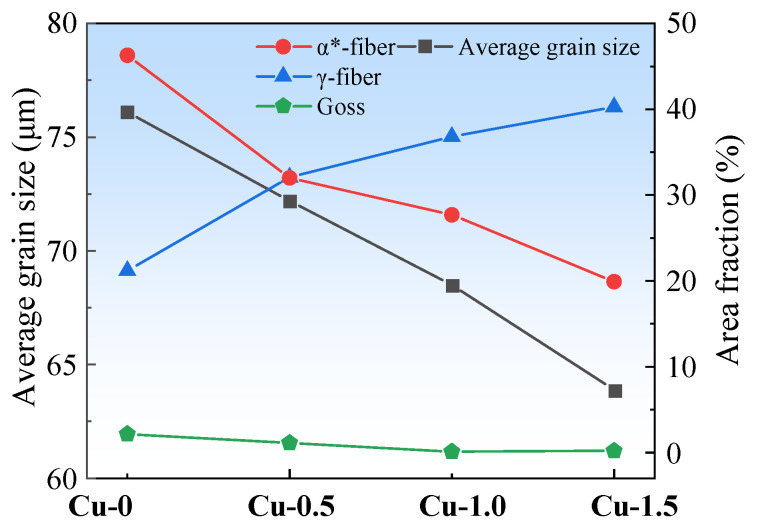
The average grain size and the area fraction of the main textures of the annealed sheets.

Figure 7 presents the average grain size and area fractions of the primary textures in annealed sheets. The right axis represents the area fraction percentages of α*-fiber, γ-fiber, and Goss textures. Increasing Cu content consistently raises the area fraction of the unfavorable γ-grains while significantly reducing that of the favorable α*-grains. In the Cu-0 annealed sheet, the area fraction of α*-grains is the largest, at 46.3%, and the area fraction of γ-grains is the smallest, at 21.19%. In contrast, in the Cu-1.5 annealed sheet, the area fraction of γ-grains is 40.28%, while the area fraction of α*-grains is 19.92%.

The growth of recrystallized grains during the annealing stage is generally closely related to the driving force for growth and boundary migration rate, while recrystallization texture evolution depends primarily on the growth kinetics of new nuclei. The grain size of normalized sheets gradually decreases with increasing Cu content. In the Cu-0 normalized sheet, the grain size before cold rolling is the coarsest, and the stored energy and grain boundary density of the cold-rolled sheet are the lowest, leading to a reduced nucleation rate during recrystallization in the final annealing process, which favors the formation of coarser recrystallized grain sizes [18]. Simultaneously, during the annealing process, the nucleation rate of Cu precipitates gradually increases with Cu content. Furthermore, studies [2,9,13,19] have confirmed that during the structural transformation of nanoscale copper precipitates, the size of copper precipitates gradually increases, enhancing the pinning effect on the grain boundaries, which in turn refines the recrystallized grains.

In Cu-0 and Cu-0.5 cold-rolled sheets, a large number of intragranular shear bands are present, within which numerous new λ-grains nucleate. During the early stages of recrystallization, the diversity of nucleation orientation and the relatively coarse deformed grain cause the rapid growth of α-grains and λ-grains. However, due to the low stored energy of α-grains and λ-grains, recrystallization is slow, and these grains are difficult to be swallowed by adjacent new recrystallized grains in the later stages. Some α-grains transform into α*-grains, which may be related to the growth advantage of {114} <481> grains, which have the high boundary mobility and driving force for grain growth. In Cu-1.0 and Cu-1.5 cold-rolled sheets, new γ-grains nucleate within deformed γ-grains and at their boundaries during the early stages of recrystallization. Subsequently, in the later stages of recrystallization, γ-grains, which have a high stored energy and numerical advantage, rapidly grow by swallowing adjacent α-grains and λ-grains with lower stored energy [17,20]. As the Cu content increases, the combined effect of increased grain boundary density and a decreased number of shear bands leads to a gradual increase in the area fraction of γ-fiber and a gradual decrease in the area fraction of α*-fiber.

### 3.2. Magnetic Properties

**Figure 8:**
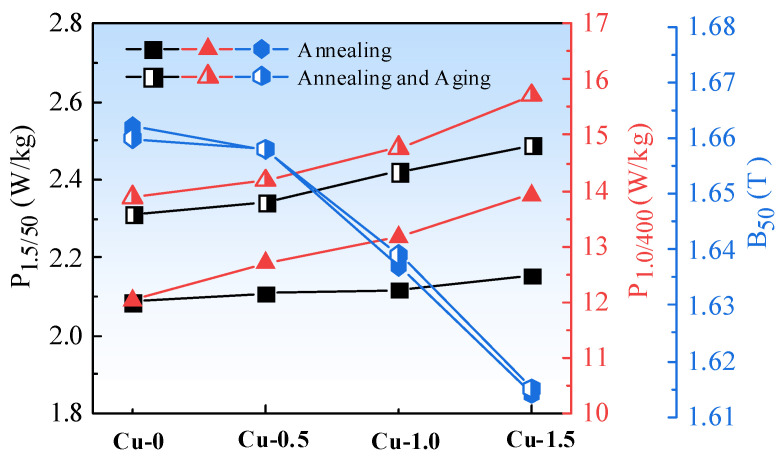
Magnetic properties of the experimental steels containing Cu before and after aging.

The magnetic properties of the annealed sheets before and after aging are shown in Figure 8. As the Cu content in the annealed sheets increases, both low-frequency iron loss (P_1.5/50_) and high-frequency iron loss (P_1.0/400_) show an increasing trend, although the increase in P_1.5/50_ is minimal. Before aging treatment, Cu-0 steel exhibits the lowest core loss, with a P_1.5/50_ value of 2.087 W/kg, while Cu-1.5 steel has the highest P_1.5/50_ value of 2.153 W/kg. The P_1.0/400_ of the annealed sheets increases with Cu content, with the P_1.0/400_ value rising from 12.026 W/kg to 13.930 W/kg. Magnetic induction (B_50_) declines significantly with increasing Cu content. The B_50_ value of Cu-0 steel is the highest at 1.662 T, whereas the B_50_ value of Cu-1.5 steel is the lowest at 1.614 T.

After aging treatment of the annealed sheets at 550 °C for 20 min, both P_1.5/50_ and P_1.0/400_ values increased, while the B_50_ value remained almost unchanged. A previous study [21] indicates that the recrystallization temperature of non-oriented silicon steel is approximately 680–720 °C, and the aging treatment process at 550 °C is relatively low. During this aging process, the microstructure and texture of the annealed sheets remain change, and the overall magnetic properties of the aged sheets slightly decrease. This is mainly due to changes in the size and number density of Cu precipitates.

The total iron loss (P_t_) in silicon steel is composed of hysteresis loss (P_h_), eddy current loss (P_e_), and anomalous loss (P_a_). Factors influencing P_h_ and the B_50_ include grain size, texture, and precipitates. As the Cu content increases, the grain size gradually decreases, and the number of grain boundaries increases, leading to higher resistance to domain wall movement and domain rotation at the grain boundaries. Consequently, P_h_ increases as the grain size decreases. Additionally, the {111} crystal plane, known for its difficulty in magnetization, exhibits a higher proportion of {111} texture as Cu content increases, resulting in a rise in P_h_ and a decrease in B_50_. With respect to precipitates, precipitates such as MnS and AlN pin the domain walls or grain boundaries during magnetization, creating resistance to domain rotation and domain wall movement. However, in non-oriented silicon steel, the Cu precipitates are nanometer-sized and much smaller than the thickness of the domain wall (30–100 nm), so Cu precipitates have a minimal impact on P_h_ [22]. P_e_ can be derived using the classical formula based on Maxwell’s equations [23]:

(1) $$P_{e}=\frac{1}{6}\frac{\pi^{2}t^{2}f^{2}B_{m}^{2}k^{2}}{\gamma \rho }\times 10^{-3}$$

where *t* is the thickness of the sheet (in mm), *f* is the frequency of the magnetic field (in Hz), *B*_m_ is the maximum magnetic induction (in Tesla), *k* is the waveform coefficient (for a sine wave, *k* = 1.11), *γ* is the density of the sheet, and *ρ* is the electrical resistivity (in Ω·mm^2^/m).

When the sheet thickness, frequency, density, and maximum magnetic induction are kept constant, the eddy current loss is generally inversely proportional to the electrical resistivity. The measured electrical resistivities of Cu-0, Cu-0.5, Cu-1.0, and Cu-1.5 experimental steels are 61.85, 60.57, 58.92, and 55.75 Ω·mm^2^/m, respectively. The electrical resistivity of the experimental steels decreases gradually. Consequently, the P_e_ increases as the Cu content rises. As the Cu content increases, the grain size of the annealed sheets decreases, resulting in higher resistance to domain movement. Moreover, the area fraction of {111} texture increases, and the combined effect of these factors leads to an increase in P_h_. On the other hand, P_a_ is mainly influenced by the domain structure and residual stress, and its variation is not significant. Changes in resistivity, grain size, and texture ultimately increase P_1.5/50_ and P_1.0/400_ [24].

### 3.3. Strengthening Mechanism

**Figure 9:**
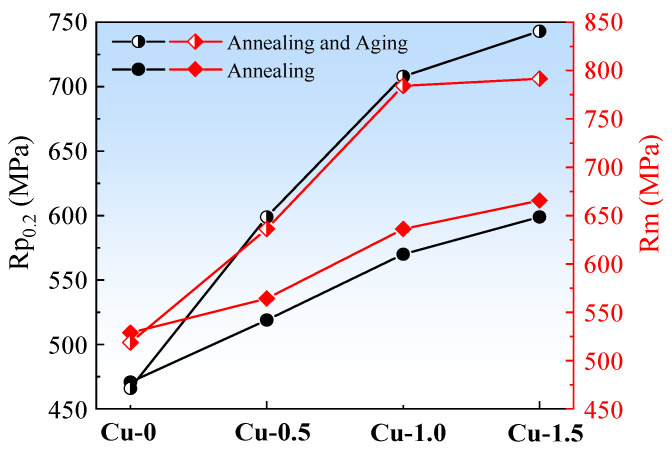
Mechanical properties of the experimental steels containing Cu before and after aging.

**Figure 10:**
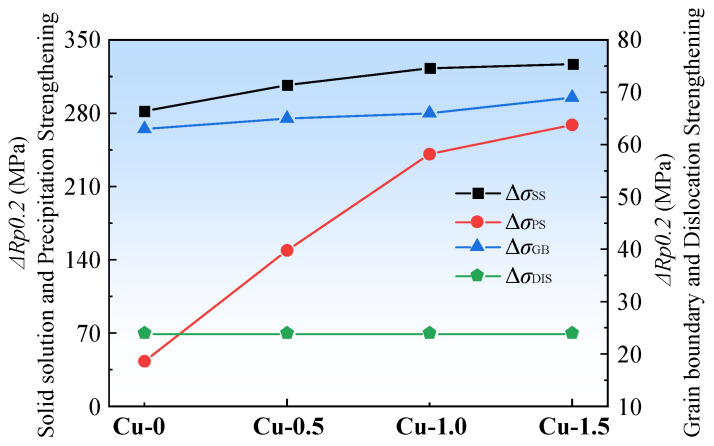
Contribution of the different strengthening mechanisms to the yield strength.

The mechanical properties of the non-oriented silicon steel annealed sheets before and after aging are shown in Figure 9. Before aging, the tensile strength (R_m_) and yield strength (R_p0.2_) of Cu-0 are significantly lower than those of the other samples, with R_m_ and R_p0.2_ being 529 MPa and 471 MPa, respectively. As the Cu content increases, both R_m_ and R_p0.2_ show an increasing trend. When the Cu content reaches 1.5%, R_m_ and R_p0.2_ reach the highest values of 665 MPa and 599 MPa, respectively. Studies [12,24,25] have shown that the solid solution strengthening effect of 1% Cu in steel is 40 MPa. Since Cu easily solid-solutes into the matrix during the annealing process of non-oriented silicon steel, a small amount precipitates during the cooling process after annealing, resulting in a significant increase in material strength [26]. As shown in Figure 10, it can be observed that for every 0.5% increase in Cu content, the yield strength increases by approximately 43 MPa. This suggests that Cu precipitation during the cooling process contributes to the enhancement of yield strength.

After aging treatment at 550 °C for 20 min, both R_m_ and R_p0.2_ increased significantly. The yield strength increased by 15.41%, 24.21%, and 24.04%, while the tensile strength increased by 12.76%, 23.26%, and 18.93%, respectively. For steels containing ≤ 1.0% Cu, the contributions from solid solution strengthening, grain boundary strengthening, and precipitation strengthening for 0.5% Cu total 121 MPa. When the copper content exceeded 1.0%, the contribution of the aging treatment to the strength is noticeably higher than that of low-Cu content steels after aging.

To clarify the mechanism of the aging treatment on the strength of Cu-bearing non-oriented silicon steel, a theoretical analysis of the yield strength increment is conducted based on various strengthening mechanisms. In ferritic steels, the yield strength primarily consists of the friction stress *σ*_0_ (54 MPa) of the ferrite matrix, solid solution strengthening of alloying elements, and grain boundary strengthening. Additionally, after annealing, the dislocation density in the final sheet decreases significantly, so the contribution of dislocation strengthening is minimal. In summary, the total yield strength and the increments generated by each strengthening mechanism can be expressed as follows [12,24,25]:

(2) $$YS=\Delta \sigma_{0}+\Delta \sigma_{SS}+\Delta \sigma_{GB}+{\left(\Delta \sigma_{DIS}^{2}+\Delta \sigma_{PS}^{2}\right)}^{1/2}$$

(3) $$\Delta \sigma_{SS}=360.36\left[C\right]+354.2\left[N\right]+83.1\left[Si\right]+32.34\left[Mn\right]+40\left[Cu\right]+470\left[P\right]$$

(4) $$\Delta \sigma_{GB}=17.402d^{-1/2}$$

(5) $$\Delta \sigma_{DIS}=\alpha MGb\rho^{1/2}$$

where *YS* represents the yield strength (in MPa), Δ*σ*_0_ refers to the friction stress of pure iron (in MPa), Δ*σ*_SS_ is the contribution of solid solution strengthening (in MPa), Δ*σ*_GB_ is the contribution of grain boundary strengthening (in MPa), Δ*σ*_DIS_ is the contribution from dislocation strengthening (in MPa), and Δ*σ*_PS_ is the contribution from precipitation strengthening (in MPa). [*C*], [*N*], [*Si*], [*Mn*], [*Cu*], and [*P*] represent the weight percentages of the respective elements. *d* denotes the average grain size of the annealed sheet (in μm), *α* is a constant (=0.435), *M* is the Taylor factor (=2.75), *G* is the shear modulus (=80.3 GPa), *b* is the Burgers vector (=0.248 nm), and *ρ* represents the dislocation density (=10^5^ m^−2^).

Figure 10 illustrates the contributions of different mechanisms to the yield strength of non-oriented silicon steel. The left axis represents the contributions of solid solution strengthening and precipitation strengthening, while the right axis represents the contributions of grain boundary strengthening and dislocation strengthening. Solid solution strengthening and precipitation strengthening serve as the primary strengthening mechanisms, providing substantial increments to the overall strength. After complete recrystallization and aging treatment, the dislocation density is extremely low, and the grain size variation is small in the sheet, so the contributions from grain boundary strengthening and dislocation strengthening remain nearly constant. After aging treatment, the Cu-0 sheet, with almost no Cu precipitates, exhibits the lowest precipitation strengthening contribution of 43 MPa. In contrast, the Cu-1.5 sheet demonstrates the highest contribution from precipitation strengthening at 269 MPa due to its substantial copper precipitation. Notably, the difference in precipitation strengthening contributions between the Cu-1.0 and Cu-0.5 sheets after aging is approximately 100 MPa. However, as the Cu content increases to 1.5%, the precipitation strengthening contribution curve flattens, suggesting that this change relates to alterations in the Cu precipitates’ structure.

### 3.4. Copper Precipitates

**Figure 11:**
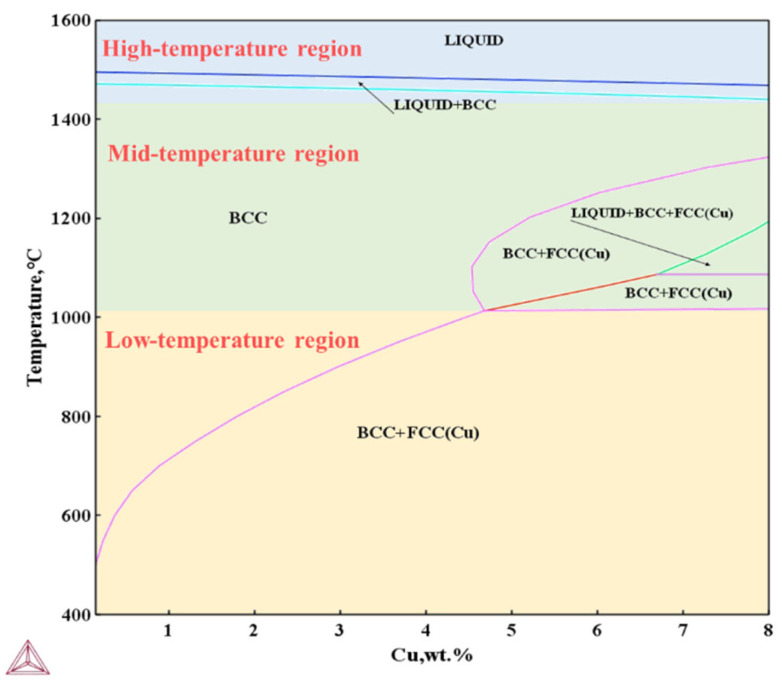
Equilibrium phase diagram of Fe-Cu-3.0% Si-0.8% Al-0.5% Mn experimental steel.

To further analyze the effect of aging treatment on the Cu precipitates in non-oriented silicon steel, Thermo-Calc 2021a software is used to calculate the variation in the form of Cu within the Fe-3.0% Si-0.8% Al-0.5% Mn non-oriented silicon steel matrix as a function of temperature, as shown in Figure 11. The phase diagram is divided into three regions: the high-temperature region (>1443 °C), the mid-temperature region (1016–1323 °C), and the low-temperature region (<1016 °C). In the high-temperature region, the matrix consists of a liquid phase and δ-Fe with a BCC structure, where Cu atoms are fully dissolved in δ-Fe. When the Cu content is below 4.67%, the matrix in the mid-temperature region is α-Fe, with Cu atoms dissolved in α-Fe, exhibiting a BCC structure. When the Cu content exceeds 6.72%, the matrix is primarily α-Fe, and a liquid phase appears. At this point, the Cu precipitate in the equilibrium state is ε-Cu with an FCC structure. In the low-temperature region, the matrix is α-Fe, and as the temperature decreases, the solubility of Cu in α-Fe decreases parabolically. At 497 °C, copper almost completely precipitates in the form of Cu-rich particles. For the non-oriented silicon steel in this study, with Cu content ranging from 0 to 1.5% and an aging temperature of 550 °C, the Cu precipitates in the final equilibrium state primarily exist in the form of ε-Cu precipitates with an FCC structure.

**Figure 12:**
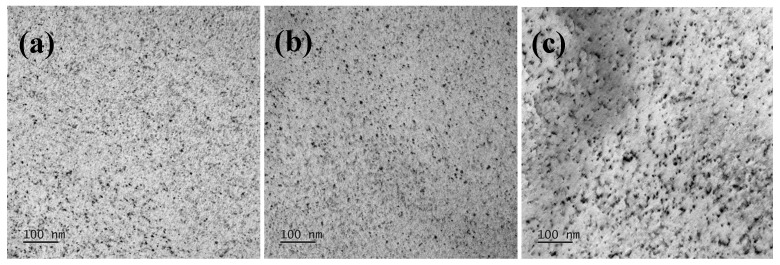
Morphology and distribution of Cu precipitates in the experimental steels after aging. (**a**) Cu-0.5, (**b**) Cu-1.0, and (**c**) Cu-1.5.

When utilizing copper element precipitation strengthening, the strengthening mechanism and final strengthening effect are influenced due to the size, number, and crystal structure of the precipitates [21]. TEM is used to detect the precipitates in the aged sheets, and the morphology and distribution of the precipitates are shown in Figure 12. After aging treatment, abundant dispersed nanoscale Cu-rich precipitates are observed in the Cu-0.5 and Cu-1.0 aged sheets. Statistical analysis using Nano Measurer 1.2 software reveals average precipitate diameters of 1.27 nm and 2.71 nm for the Cu-0.5 and Cu-1.0 sheets, respectively. Conversely, the Cu-1.5 aged sheet exhibits clustered precipitates that progressively coarsen, with partial transition from spherical to ellipsoidal morphology, resulting in an average precipitate size of 13.28 nm. Consequently, with the increase in copper content in non-oriented silicon steel, the dispersed nanoscale precipitates in the annealed sheets undergo growth and coarsening during the aging process, accompanied by microstructural evolution.

**Figure 13:**
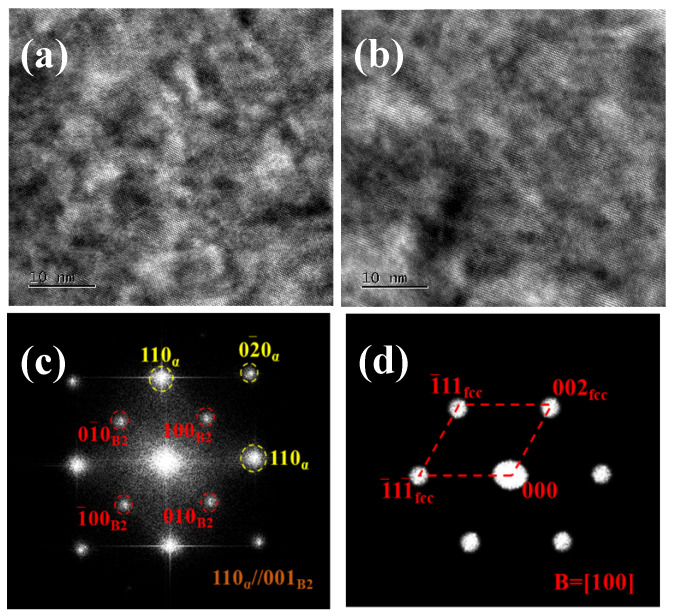
High-resolution images and Fourier transformation of the high-resolution images of the precipitates in aged sheets. (**a**,**c**) Cu-1.0, (**b**,**d**) Cu-1.5.

Figure 13a,b shows high-resolution transmission electron microscopy (HR-TEM) images of the precipitates in the Cu-1.0 and Cu-1.5 aged sheets, respectively. It can be observed that the Cu-rich precipitates are fully coherent with the non-oriented silicon steel matrix. Figure 13c presents the HR-TEM image of the Cu-1.0 aged sheet along with its corresponding Fourier transform (FFT) and diffraction pattern calibration. The FFT image displays superlattice spots, identifying the precipitate as a B2-ordered structure, where iron atoms at body-centered positions are replaced by Cu atoms. The orientation relationship between the precipitate and the matrix is [001]_α_//[001]_B2_. Figure 13d shows the HR-TEM image of the Cu-1.5 aged sheet, along with its corresponding FFT and diffraction pattern calibration. In this case, the precipitate phase is ε-Cu with a face-centered cubic (FCC) structure.

Based on phase diagram calculations and experimental results, it is inferred that the crystal structure of the copper precipitate transitions from B2-Cu to FCC-Cu. At an aging temperature of 550 °C, the solubility of Cu in α-Fe is relatively low. When the Cu content is 1.0%, the Cu-rich precipitates exhibit a metastable B2 structure, which is coherent with the matrix. At this stage, the size and spacing of the coherent precipitates are small, and both the interface energy and coherent strain energy are low. As the Cu content increases, the coherent strain energy increases, and the structure of the Cu precipitate ultimately transforms into the stable ε-Cu phase with an FCC structure [12].

The size, number density, and crystal structure of the nanoscale Cu precipitates significantly impact their contribution to precipitation strengthening. Based on differences in precipitate size, the dislocation interaction mechanisms in the matrix are categorized into the cutting mechanism and the bypass mechanism [27]. In the Cu-1.0 aged sheet, the B2 Cu-rich precipitates have an average size of 2.71 nm. In this case, dislocations cut through the nanoscale precipitates via slip, and the cutting mechanism dominates the precipitation strengthening. According to the quantitative calculations from reference [28], under the cutting mechanism, modulus strengthening and coherent strengthening contribute primarily. Therefore, the strength of the Cu-1.0 aged sheet is maximized through the cutting mechanism. In the Cu-1.5 aged sheet, the Cu-rich precipitates grow to 13.28 nm, and the precipitate structure transitions to FCC. When the precipitate size reaches the critical size, dislocations bypass the non-shearable precipitates by bending and leaving dislocation loops. At this point, the bypass mechanism dominates the precipitation strengthening.

Consequently, increasing the Cu content in non-oriented silicon steel and subjecting it to a 550 °C aging treatment for 20 min after annealing enhances the yield strength of the non-oriented silicon steel by 149–269 MPa due to the precipitation of Cu-rich precipitates. The variation in Cu content leads to changes in the size and structure of the precipitates during the aging process. Specifically, the precipitation process involves the dissolution of smaller precipitates, their subsequent growth, and the structural transformation of larger precipitates. Ultimately, Cu precipitates with different structures contribute to varying increments in yield strength through two strengthening mechanisms via the cutting mechanism and the bypass mechanism.

## 4. Conclusions

(1) Increasing the copper content in 3.0% Si-0.8% Al-0.5% Mn non-oriented silicon steel leads to a noticeable reduction in the grain size of the normalized sheet and a decrease in the area fraction of the α*-fiber. Simultaneously, the area fraction of the γ-fiber significantly increases. In the 0.20 mm-thick cold-rolled sheet, the shear band content is reduced. For the annealed sheet, the grain size is reduced, which promotes an increase in the area fraction of the γ-fiber while suppressing the development of λ-fiber and α*-fiber.
(2) The addition of Cu deteriorates the magnetic properties, with the P_1.5/50_, P_1.0/400_, and B_50_ values for Cu-0 steel being 2.087 W/kg, 12.026 W/kg, and 1.662 T, respectively. For Cu-1.5 steel, the corresponding values are 2.153 W/kg, 13.930 W/kg, and 1.614 T. The solid solution strengthening and partial precipitation strengthening effects of 0.5% Cu lead to an increase in R_p0.2_ by 43 MPa. For Cu-1.5 steel, the R_m_ and R_p0.2_ are 665 MPa and 599 MPa, respectively.
(3) After aging treatment at 550 °C for 20 min, the P_1.5/50_ and P_1.0/400_ values slightly increase, while the B_50_ value remains almost unchanged. The R_p0.2_ values for Cu-0.5, Cu-1.0, and Cu-1.5 increase by 15.41%, 24.21%, and 24.04%, respectively. When the Cu content is ≤1.0%, the difference in contributions from solid solution strengthening, grain boundary strengthening, and aging precipitation strengthening for 0.5% Cu is 121 MPa.
(4) After aging treatment, the average sizes of Cu-rich precipitates in Cu-1.0 and Cu-1.5 are 2.71 nm and 13.28 nm, respectively, and the crystal structure changes from B2-Cu to FCC-Cu. The addition of 1.0% and 1.5% Cu enhances the yield strength of non-oriented silicon steel by 241–269 MPa through the cutting and bypass mechanisms of Cu-rich precipitates.

## Figures and Tables

**Table 1:** Chemical composition of the experimental steels containing Cu (wt%).

Samples	C	Si	Als	Mn	Cu	P	S	N	Fe
Cu-0	0.0021	3.076	0.7959	0.511	0.021	0.015	0.0024	0.0020	Bal.
Cu-0.5	0.0019	3.186	0.8059	0.525	0.451	0.012	0.0025	0.0017	Bal.
Cu-1.0	0.0024	3.079	0.7563	0.570	1.006	0.015	0.0026	0.0021	Bal.
Cu-1.5	0.0025	2.926	0.7975	0.554	1.469	0.011	0.0024	0.0019	Bal.

## Data Availability

The data presented in this study are available on request from the corresponding author due to confidentiality requirements related to commercial project data.
